# Supplementary material for: Identification of photoperiod-induced specific miRNAs in the adrenal glands of Sunite sheep (Ovis aries)
Source: Front Vet Sci. 2022 Jul 22;9:888207. doi: 10.3389/fvets.2022.888207 (PMC9354845; doi:10.3389/fvets.2022.888207)
Supplement: Supplementary file 1 [file Data_Sheet_1.ZIP › Supplementary file/miRDeep2 file.zip!/miRDeep2 file/Know_Structure/SPLP42A1/oar-mir-221.pdf]

miRBase precursor : oar-mir-221
Total read count : 2805
oar-miR-221 read count : 2566
remaining reads : 239

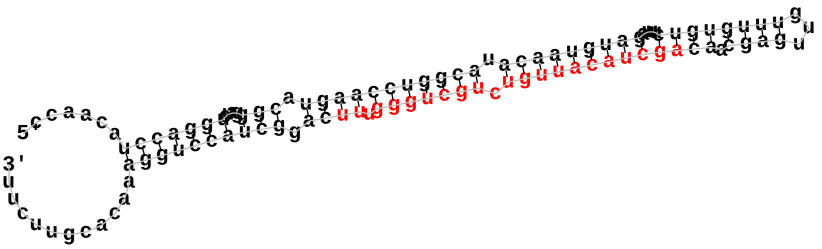

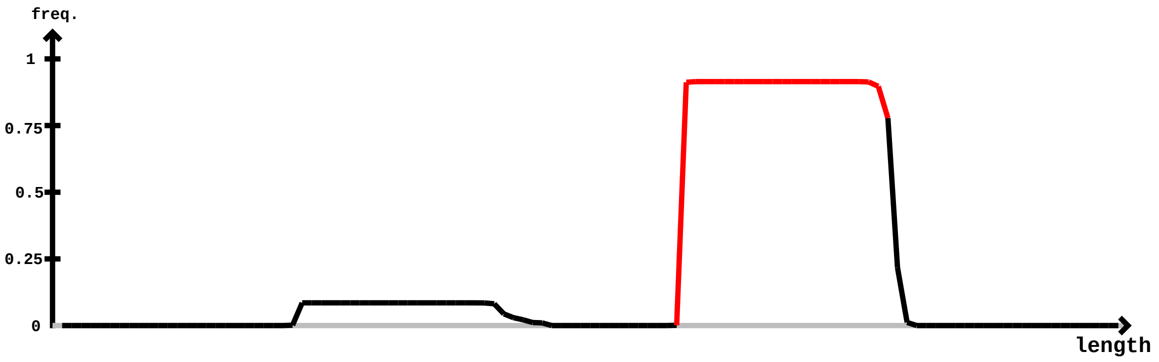


```
                                                    oar-miR-221
5'- ccaacauccaggucuagggcaugaaccuggcauacaauguagauuucuguguuuguugagcaacagcuacauugucugcuggguuucaggcuaccuggaaacacguucuu   -3'    exp
    ......(((((((....(((.(((((((((((.(((((((((....((((((((...)))).))))))))))))).))))))).)))).))))))))))...........   reads      mm         sample
    .......................aaccuggcauacaauguagauu.................................................................   2          0          seq
    .......................aaccuggcauacaauguagauuu................................................................   2          0          seq
    ........................accuggcauacaauguaga...................................................................   1          0          seq
    ........................accuggcauacaauguagau..................................................................   9          0          seq
    ........................accuggcauacaauguagauu.................................................................   104        0          seq
    ........................accuggcauacaauguagauuu................................................................   38         0          seq
    ........................accuggcauacaauguagauuuc...............................................................   22         0          seq
    ........................accuggcauacaauguagauuucu..............................................................   29         0          seq
    ........................accuggcauacaauguagauuucug.............................................................   4          0          seq
    ........................accuggcauacaauguagauuucugu............................................................   28         0          seq
    ...............................................................cagcuacauugucugcuggguuu........................   2          0          seq
    ...............................................................cagcuacauugucugcuggguuuc.......................   1          0          seq
    ................................................................agcuacauugucugcuggg...........................   4          0          seq
    ................................................................agcuacauugucugcugggu..........................   45         0          seq
    ................................................................agcuacauugucugcuggguu.........................   332        0          seq
    ................................................................agcuacauugucugcuggguuu........................   1569       0          seq
    ................................................................agcuacauugucugcuggguuuc.......................   573        0          seq
    ................................................................agcuacauugucugcuggguuuca......................   33         0          seq
    .................................................................gcuacauugucugcugggu..........................   1          0          seq
    .................................................................gcuacauugucugcuggguu.........................   2          0          seq
    .................................................................gcuacauugucugcuggguuu........................   2          0          seq
    .................................................................gcuacauugucugcuggguuuc.......................   2          0          seq
```
